# Supplementary material for: Horizontal Combination of MEK and PI3K/mTOR Inhibition in BRAF Mutant Tumor Cells with or without Concomitant PI3K Pathway Mutations
Source: Int J Mol Sci. 2020 Oct 16;21(20):7649. doi: 10.3390/ijms21207649 (PMC7589607; doi:10.3390/ijms21207649)
Supplement: Supplementary file 1 [file ijms-21-07649-s001.pdf]

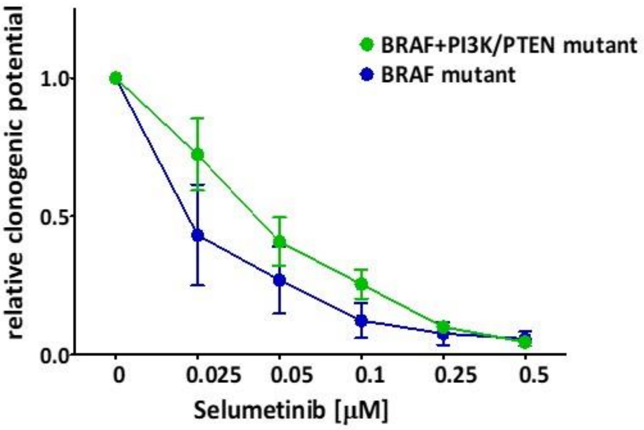

Figure S1. Average clonogenic potential in the mutational groups after treatment with selumetinib, without colon cancer cell line data. Data is shown the mean ± SEM from at least three independent experiments.

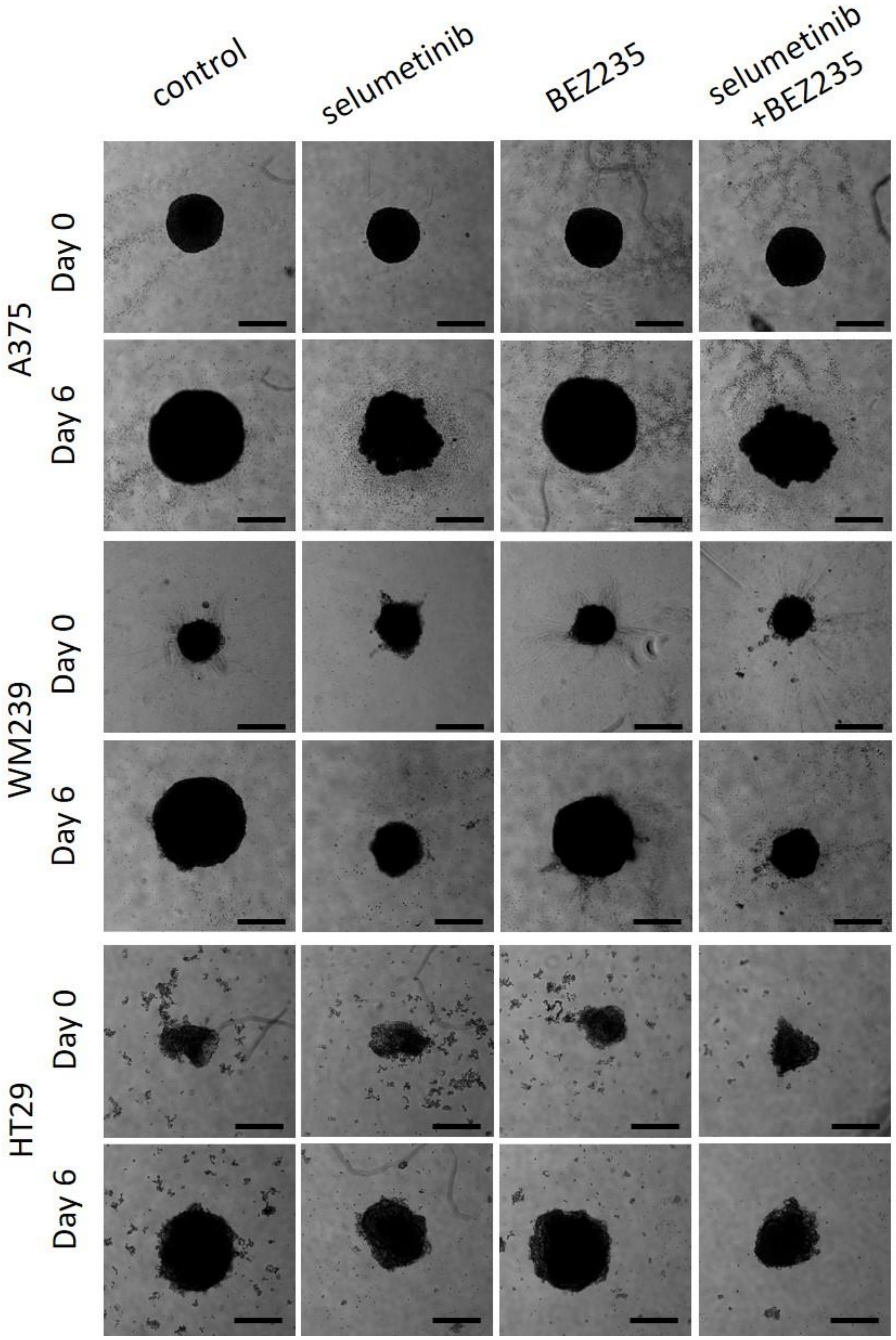

Figure S2. Representative pictures of A375, WM239 and HT29 spheroids at the beginning (Day 0) and at the end (Day 6) of the experiment. Scale bar means 500 μm.

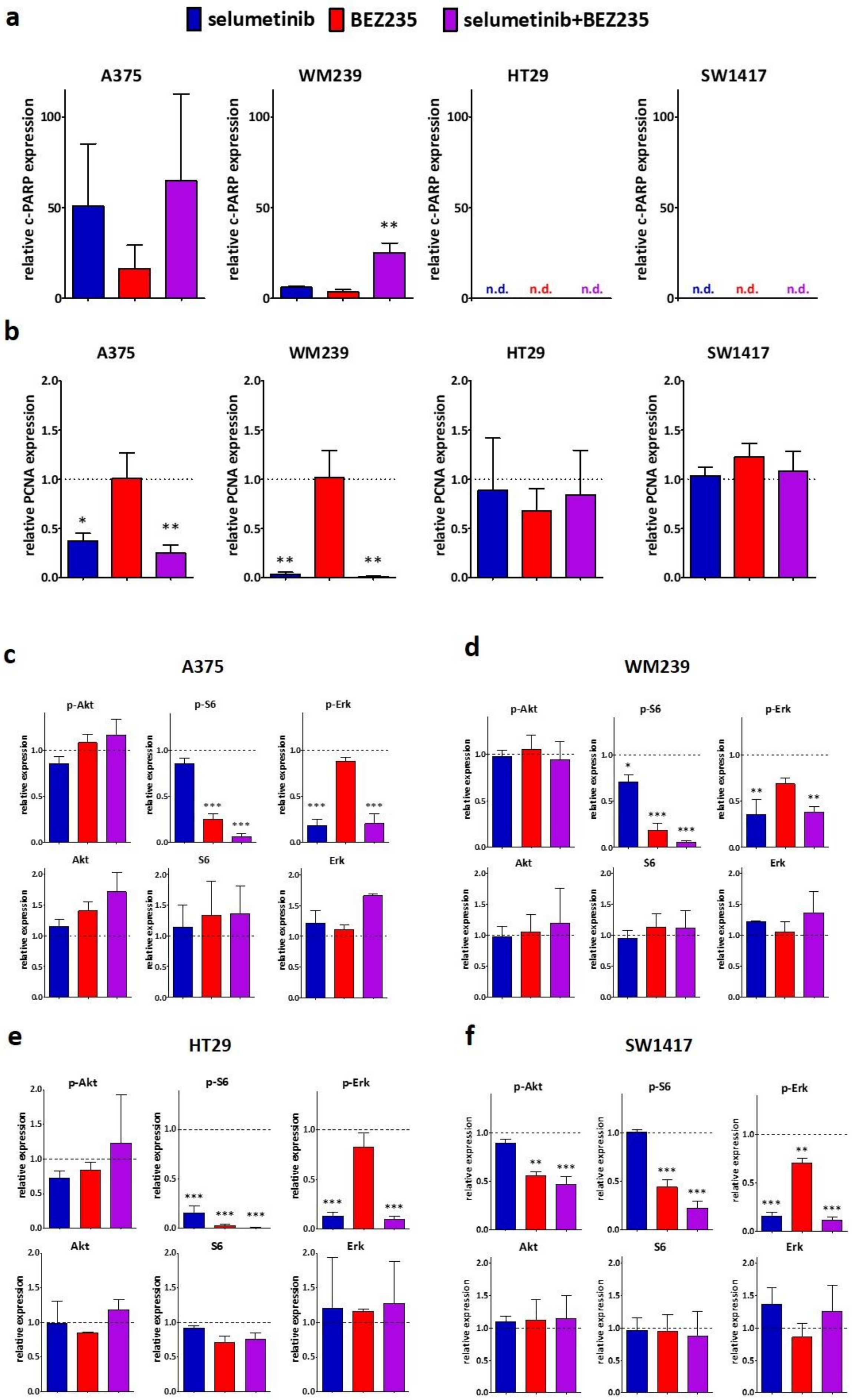

a
selumetinib
BEZ235
selumetinib+BEZ235
A375
WM239
HT29
SW1417
relative c-PARP expression
n.d.
b
relative PCNA expression
c
A375
d
WM239
e
HT29
f
SW1417
p-Akt
p-S6
p-Erk
Akt
S6
Erk
relative expression

Figure S3. Quantification of the immunoblots. (**a**–**b**) Cleaved-PARP expression was detected in A375 and WM239 cells and was significantly higher upon treatment with combination in WM239 line. PCNA expression decreased upon treatment with selumetinib and combination in A375 and WM239 cells and was significant in A375 upon combination treatment. Data is shown as average ± SEM from three independent experiments. (n.d. = not detected) (**c**–**f**) Significant decrease of p-S6 level was detected upon treatment with combination in all the cells. Furthermore, in the SW1417 cell line, Akt and Erk phosphorylation was also decreased significantly upon treatment with combination (**f**). In addition, single selumetinib treatment was able to decrease Erk protein activation only in case of SW1417 cell line. Asterisks mean a significant difference between the treatment groups and the control by * $p < 0.05$, ** $p < 0.01$ and *** $p < 0.001$.

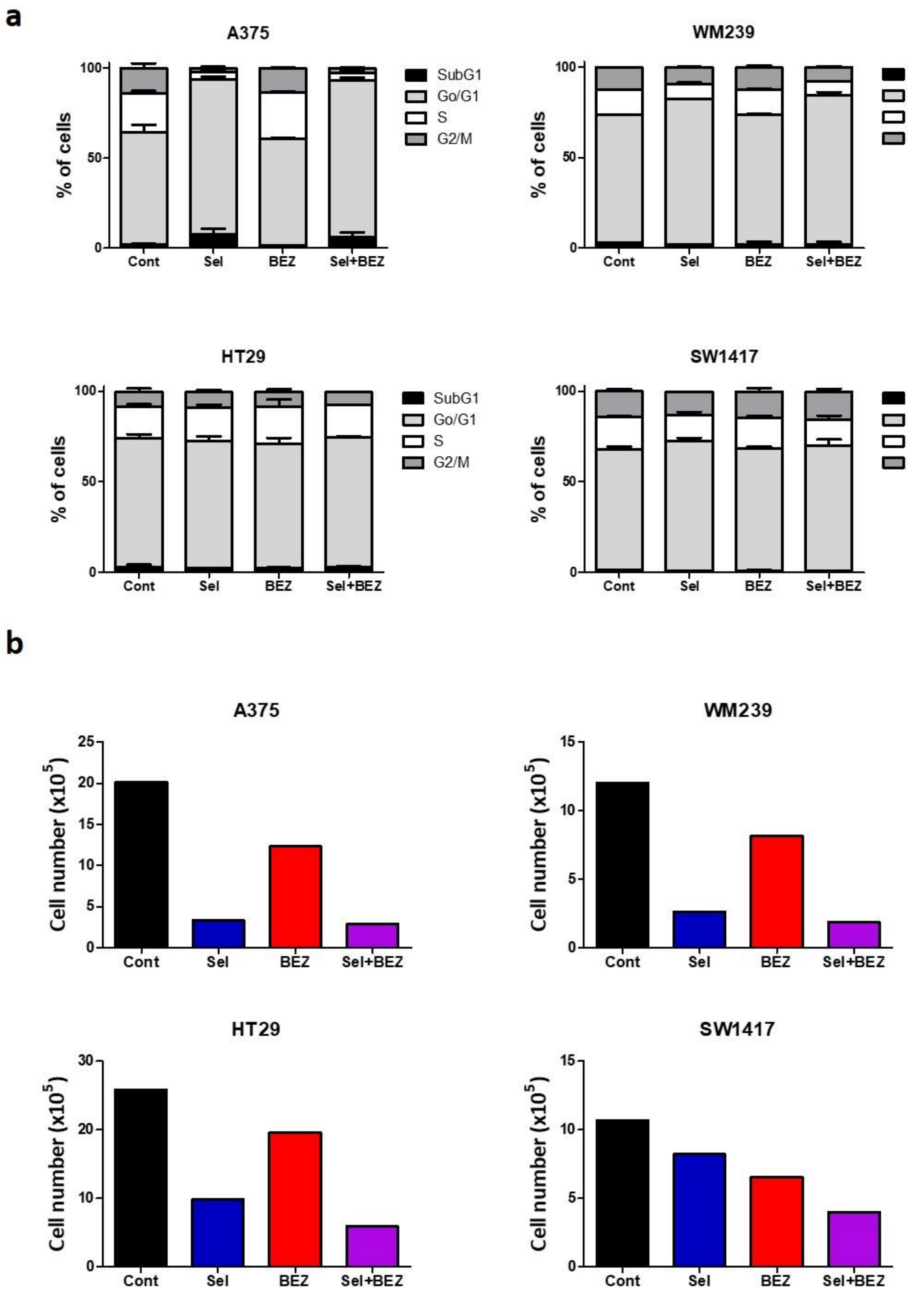

Figure S4. Cell cycle and cell number analysis. (**a**) Cell cycle analysis after treatment with 100nM selumetinib (Sel), 10nM BEZ235 (BEZ) or both (Sel + BEZ) for 72 hours. Increased ratio of cells in subG1 phase was detected in A375 cells after treatment with selumetinib or combination. Decreased proportion of cells in S phase was measured in WM239 cells after selumetinib or combination treatments. Data is shown the mean ± SD from two independent experiments. (**b**) Total cell number after treatment with 100nM selumetinib (Sel), 10nM BEZ235 (BEZ) or both of them (Sel + BEZ) for 72 hours. The combination treatment induced the strongest cell number decrease after combination treatments in HT29 and SW1417 cells.

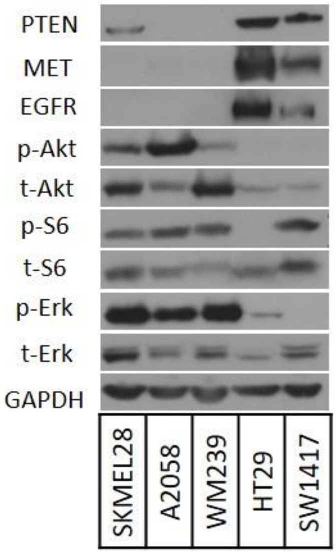

Figure S5. Representative immunoblots of five double mutant cell lines. MET and EGFR was detected in colon cell lines (HT29, SW1417). *p*-Akt and *p*-Erk level was enhanced in PTEN mutant cells (SKMEL28, A2058, WM239). *p*-S6 expression was the lowest in HT29 compare to the other cells. Blots are representative pictures from three independent experiments.

Table S1. IC50 values after treatment with the indicated inhibitors for 72 h.

IC50	A375	WM35	CRL5885	SKMEL28	A2058	WM239	HT29	SW1417
Selu (µM)	0.05	0.09	0.23	0.17	0.58	0.14	2.03	>5
BEZ235 (nM)	62.4	10.3	25.2	19.3	9.0	32.4	26.4	16.1
